# Rationale for the design of an oncology trial using a generic targeted therapy multi-drug regimen for NSCLC patients without treatment options (Review)

**DOI:** 10.3892/or.2013.2631

**Published:** 2013-07-22

**Authors:** STEFAN LANGHAMMER

**Affiliations:** Life Science Consulting, Schloss Pesch, D-40668 Meerbusch, Germany

**Keywords:** targeted therapy drugs, drug resistance, multi-drug regimen, HIV, highly active antiretroviral therapy

## Abstract

Despite more than 70 years of research concerning medication for cancer treatment, the disease still remains one of the leading causes of mortality worldwide. Many cancer types lead to death within a period of months to years. The original class of chemotherapeutics is not selective for tumor cells and often has limited efficacy, while treated patients suffer from adverse side-effects. To date, the concept of tumor-specific targeted therapy drugs has not fulfilled its expectation to provide a key for a cure. Today, many oncology trials are designed using a combination of chemotherapeutics with targeted therapy drugs. However, these approaches have limited outcomes in most cancer indications. This perspective review provides a rationale to combine targeted therapy drugs for cancer treatment based on observations of evolutionary principles of tumor development and HIV infections. In both diseases, the mechanisms of immune evasion and drug resistance can be compared to some extent. However, only for HIV is a breakthrough treatment available, which is the highly active antiretroviral therapy (HAART). The principles of HAART and recent findings from cancer research were employed to construct a hypothetical model for cancer treatment with a multi-drug regimen of targeted therapy drugs. As an example of this hypothesis, it is proposed to combine already marketed targeted therapy drugs against VEGFRs, EGFR, CXCR4 and COX2 in an oncology trial for non-small cell lung cancer patients without further treatment options.

## 1. Overview of the mechanisms of anticancer drugs

Drugs for cancer treatment can be classified into the categories of chemotherapeutic drugs and targeted therapy drugs. Examples of chemotherapeutic drugs include alkylating or alkylating-like agents such as capecitabine, mitotic inhibitors such as paclitaxel and topoisomerase inhibitors such as irinotecan. All of these drugs act as cytotoxic or cytostatic agents by killing rapidly dividing cells.

Targeted therapy agents include endocrine therapy drugs such as tamoxifen, antigrowth factor drugs such as the monoclonal antibody trastuzumab against Her2 or the receptor tyrosine kinase inhibitor (RTKI), gefitinib against the epidermal growth factor receptor (EGFR) and anti-angiogenesis drugs such as the monoclonal antibody bevacizumab against vascular endothelial growth factor (VEGF) or the RTKI sunitinib against VEGF receptors (VEGFRs) (1). All of these drugs act by specifically blocking signal transduction pathways involved in tumor development.

Historically, chemotherapeutic drugs were the first effective agents used against malignant diseases. In the 1940s it was discovered that a derivate of a chemical warfare agent, nitrogen mustard, was effective for treating lymphoma when applied to patients intravenously (2,3). Cytotoxic chemotherapies are based on the classical ‘principles of chemotherapy’ as defined by the observance that tumors exhibit a sigmoid-shaped Gompertzian growth curve and thus cytoxic drugs are most effective in killing tumor cells within the logarithmic growth phase (4). For many cancer indications, cytotoxic chemotherapeutics are still the recommended first-line therapy. These agents include the platinum-based drugs cisplatin or carboplatin for non-small cell lung cancer (NSCLC) (5) or capecitabine in combination with oxaliplatin for colorectal cancer (6,7). This class of drugs does not differentiate between cancerous and normal cells and thus induces systemic toxicity and adverse reactions (8,9). The current survival rates of cancer patients mostly treated with cytotoxic chemotherapeutics and/or undergoing surgery and/or radiation therapy depends very much on the site of the primary tumor. Female breast *in situ*, uterine corpus and melanoma have 10-year relative survival rates of up to 100% (female breast *in situ*). In contrast the 5-year survival rate of patients with NSCLC and liver cancer is ~15% and in pancreatic cancer ~5% since cytotoxic chemotherapeutics are unable to cure metastatic disease even after successful surgical tumor resection (10,11).

In contrast to cytotoxic chemotherapeutics, the concept of targeted therapy is aimed to specifically target a biological pathway that is critical for tumor development or tumor maintenance and that causes regression or destruction of the malignant process when it is inhibited (12). Endocrine therapy drugs were developed for inhibition or modulation of hormone receptors for hormone-sensitive tumors. The estrogen receptor modulator (SERM) tamoxifen was the first targeted therapy drug for anticancer treatment selectively inhibiting estrogen binding to its receptor (13). When administered as an adjuvant therapy for primary treatment of estrogen receptor (ER)-sensitive breast cancer, tamoxifen was shown to reduce relapse and mortality rates (14), and to decrease recurrence rates in ER-positive breast cancer patients by 50% 15 years after diagnosis (13).

Another example of targeted therapy drugs are inhibitors against the EGFR. EGFR is a well characterized example of a growth factor receptor which plays a central role in tumor development when becoming overexpressed and/or mutated. Overexpression and mutation of EGFR leads to proliferation, invasion of surrounding tissues, angiogenesis and distant metastasis (15). Activation of EGFR was also shown to influence resistance to cytotoxic chemotherapeutic agents. Intracellular signaling leading to all of these EGFR-mediated processes include the MAP kinase pathway, PI3K and Akt signaling. Several targeted therapy drugs have been approved by the FDA and EMEA for blocking the EGFR pathway either by binding to its ligand EGF (mAb panitumumab) or by inhibiting its tyrosine kinase activity (mAb cetuximab and RTKIs gefitinib and erlotinib) (16,17). Gefitinib (Iressa) for example is an orally available small-molecule RTKI (18) approved for the first-line therapy of NSCLC patients with activating mutations of the EGFR tyrosine kinase domain (19). In a subgroup of the INTEREST trial including patients with activating EGFR mutations, gefitinib was shown to improve the progression-free survival (PFS) but not the overall survival (OS) when compared to docetaxel. The median survival of patients under gefitinib treatment was 14.2 months compared to 7.6 months in the overall population (20).

Since the discovery of the impact of angiogenesis on tumor biology by Folkman (21) several angiogenesis targets have been confirmed, and small-molecule RTKIs and monoclonal antibodies have been approved as targeted therapies for the treatment of different types of malignancies (22,23). One of the most prominent anti-angiogenic targets is the signal transduction by the VEGF via its receptors VEGR1-3. The monoclonal antibody bevacizumab (Avastin) binds to VEGF and was the first approved anti-angiogenic therapy. Today bevacizumab is used as a first-line therapy for colorectal cancer (CRC) and metastatic renal cell carcinoma (mRCC) (24). Several small molecule RTKIs that target VEGFRs have been approved for anticancer treatment. Among them are sunitinib (Sutent) for RCC and GIST, sorafenib (Nexavar) for RCC and inoperable HCC and vandetanib (Caprelsa) for late stage medullary thyroid cancer (25). However, the development of breakthrough targeted therapies based on anti-angiogenic and anti-growth cancer treatment has been unsuccessful until recently (26,27). Prolongation of survival by targeted therapies alone or in combination with cytotoxic chemotherapeutics often can only be achieved for several months to several years. Treatment of mRCC with sunitinib improved the OS by more than 2 years compared to treatment with interferon-α (28). Bevacizumab in combination with chemotherapy was found to prolong the life of patients with metastatic CRC only by 4 to 5 months (29). Drawbacks associated with the application of this class of drugs include resistance to anti-angiogenic therapies mediated by the tumor microenvironment and stromal cells (30) and induction of tumor invasiveness (31). The most recent example of this failure is the revoke of FDA approval for the monoclonal anti-VEGF antibody bevacizumab for treatment of Her2-negative metastatic breast carcinoma at the end of 2011 due to its unfavorable risk-benefit profile. It could not be shown that bevacizumab significantly delays tumor growth or prolong the lives of women with breast cancer (32). Today, the design of clinical trials in oncology focuses on the combination of cytotoxic chemotherapeutics with targeted therapies. Biomarkers are used to stratify patients in order to predict the responsiveness for drug dose selection or to monitor therapy effectiveness of certain targeted therapy drugs (33).

## 2. Comparing the biological principles of tumor development and HIV infection

In many diseases of different origins, common underlying biological mechanisms play central roles. For example, the dysregulation of EGFR signal transduction is an important hallmark of certain malignancies as described above (17,34) and at the same time plays a critical role in poxvirus spreading (35). Inhibitors against EGFR developed for anticancer treatment are effective against poxvirus infections (36). The observation and the analysis of common mechanisms in different types of diseases provide the opportunity to draw conclusions from the treatment of one disease to another. In a holistic model, the mechanisms of an HIV-1 infection and tumor development are comparable to a certain extent. These entirely different diseases have common features that follow the principles of the Darwinian evolutionary system. HIV-1 comprises a viral genome of ~9,700 bases of single-stranded RNA (37) and replicates within an estimated average total of 10.3×10^9^ virions/day (38). Thus, the disease driver of an HIV infection is primarily the replication rate (39) in combination with a high mutation rate introduced by the lack of proof-reading mechanism of the transcriptase enzyme (40) and secondarily the genetic variability (41). In tumor cells, the conditions are exactly opposite. The genome of a malignant human cell consists of ~3.2×10^9^ base pairs, while its replication takes ~1–2 days (42,43). Thus, in tumors, the genetic heterogeneity instead of the replication rate is the primary evolutionary driver (44–47). Most importantly, in both diseases, in malignancies and in HIV infections, the immune system is unable to control the disease due to the evolutionary drivers that steer the processes of evasion from immunological responses. In HIV infections and in tumor development, these mechanisms include the generation of escape mutants and the suppression of the immune system (43,48–54). Additionally in tumor development the regulation of rescue pathways plays a critical role to evade immunological responses (55,56).

## 3. Breakthrough in HIV treatment through the prevention of viral resistance using a multi-drug regimen of targeted therapy drugs

Despite the anticipated common underlying biological mechanisms of HIV infections and tumor development, the development of an effective therapy concept against HIV was a great success while there is still no groundbreaking treatment for many types of cancer. At the beginning of the HIV epidemic, patients were treated with monotherapy of the nucleoside analogue reverse transcriptase inhibitor (NRTI) zidovudine developed in 1964. NRTIs selectively inhibit HIV reverse transcriptase and thereby specifically block transcription of viral DNA from viral RNA. Even though this treatment exhibited some effect, the rates of progression-free survival were still low. In a hemophilic cohort of 111 patients treated solely with zidovudine and followed up for 11 years, the progression rates to AIDS, symptoms and death were as high as 85% (57). In 1996, only 16 years after the identification of HIV as the cause of AIDS, the breakthrough in HIV therapy was achieved when targeted therapy drugs were applied in a combinational protocol introducing the highly active antiretroviral therapy (HAART) (58). One year later the clinical superiority of a three-drug regimen over a two-drug regimen by using the selective protease inhibitor indinavir together with the NRTIs zidovudine and lamivudine was demonstrated (59). Today, HAART consists of at least three drugs, including either a protease inhibitor or a non-NRTI (NNRTI) and two NRTIs. All three different drugs used in HAART are selective targeted therapy compounds against critical steps in the HIV-1 replication cycle. The drug class of HIV-1 protease inhibitors is among the first successful examples of highly selective targeted therapy drugs (60). A prospective cohort study evaluated the long-term effectiveness of HAART and showed a reduction in the progression to AIDS or death by 86% (61).

The successful treatment of HIV by HAART shows that it is possible to control a disease that follows an evolutionary concept similar to cancer. In both diseases, primary evolutionary factors, a high replication rate in HIV and a large genomic variability in cancer, define the route of evasion from immunological responses. HAART is successful because it is based on two key principles of treatment: i) high selectivity of drugs against disease-specific targets, thus preventing severe toxicities, adverse reactions and damage of immune system components; and ii) effective combination of disease-specific targets, thus preventing evolutionarily driven generation of escape mutants and drug resistances. Transferring these principles to cancer treatment would mean to address tumor targets as selective as possible reducing side-effects and to identify a multi-drug regimen of targeted therapy drugs for each type of malignancy preventing tumor cell rescue and drug resistance. However, at present most of the current anticancer drug therapies consist of chemotherapeutics that do not follow the principle of drug selectivity (5–7). These therapies lead to dose-dependent side-effects and adverse reactions but mostly have a modest to moderate effect on the malignant disease by prolonging survival times only by a few months to years at maximum (10). Most of these treatments have toxic effects on the hematological system and lead to opportunistic infections that have to be controlled in parallel to the chemotherapeutic treatment (8,62,63).

## 4. Implications for the design of a generic oncology trial with a multi-drug regimen using targeted therapy drugs for NSCLC patients not indicated for treatment

Lung cancer is the leading cause of cancer-related mortality worldwide. Histopathological grading identifies ~85% of lung cancers as NSCLCs and 15–20% as small-cell lung cancers (SCLCs). NSCLCs can be subdivided into squamous cell carcinoma and adenocarcinoma, accounting for 34 and 55% respectively, and into other subtypes such as large cell carcinoma (64). For NSCLC cases up to grade IIIA, the standard of care is surgical resection of the primary tumor which can be supported by adjuvant chemotherapy and/or radiotherapy. However, most often NSCLC is diagnosed at advanced stages beyond stage IIIB. These tumors are treated with first-line neoadjuvant chemotherapies followed by tumor resection or alternatively combined with radiotherapy. First-line chemotherapies against NSCLC consist of platinum-based drugs (carboplatin or cisplatin) combined with third generation cytotoxic drugs such as docetaxel, paclitaxel, irinotecan, gemcitabin, vinorelbin and pemetrexed (5). Several targeted therapy drugs have been approved for the treatment of NSCLC. These include gefitinib, approved for first line therapy of NSCLC with activating mutations of the EGFR tyrosine kinase domain (19) and bevacizumab approved for treatment of non-squamous NSCLC in combination with platinium-based therapy (65). Another targeted therapy drug is crizitonib which inhibits the EML4-ALK fusion protein, an oncogenic driver in a small percentage of NSCLC patients (66). Second-line therapies include docetaxel, erlotinib, gefitinib, pemetrexed and platinum-based therapy (5). The 5-year survival rate for NSCLC patients is only ~15% (10) and thus the medical need for the development of effective treatment concepts for NSCLC patients is one of the greatest challenges for health care systems worldwide.

For the design of a clinical trial for NSCLC patients without further treatment options based on the concept of selectivity and adequate target combination as discussed above, four signaling pathways appear to be suitable targets against which drugs are already marketed in different indications:

### VEGFR2-VEGF

VEGF signaling is a well characterized target complex with proven importance for tumor angiogenesis and tumor development, including NSCLC (27). Among the VEGF receptors VEGFR2 facilitates tumor growth by inducing angiogenesis in tumor endothelial cells (67). Evidence suggests that VEGFR2 signaling also influences the motility of tumor cells, such as in pancreatic cancer cells (68). Signal transduction by VEGFR2 is mediated through the Ras/Raf kinase pathway connected to MAP kinase signaling and the PI3K/Akt pathway inducing angiogenesis by mediating cell proliferation and cell-survival of tumor endothelial cells (69). For blocking the VEGF/VEGFR2 signaling pathway, several targeted therapy drugs are available. These include the small-molecule RTKI sunitinib and the monoclonal antibody bevacizumab. Sunitinib is a multi-targeted tyrosine kinase inhibitor against VEGF receptors that also inhibits the activity of other tyrosine kinases shown to be involved in tumor growth (70). Bevacizumab is directed against VEGF and blocks binding of this ligand to VEGF receptors (71). Tumors that have been treated with selective VEGFR2 inhibitors develop hypoxic microenvironments by a compromised blood supply. It was shown that this effect is countered by upregulation of growth factors which have the capacity to replace VEGF and stimulate new blood vessel growth such as EGF and SDF1α as well as their receptors (30).

### CXCR4-SDF1α

The observation that treatment resistance to the blockage of VEGFR2 is mediated by EGF and SDF1α and their receptors qualifies these pathways as further potential targets for a multi-drug-regimen targeted therapy for NSCLC treatment. SDF1α and its receptors CXCR4 and CXCR7 were previously shown to be involved in tumor development and tumor metastasis (72–74). SDF1α signal transduction through CXCR4 is a well described pathway that leads to activation of JAK/Stat, MAPK/ERK and PI3K with phosphorylation of Akt (75,76). CXCR4 activation plays a role in tumor metastasis, induction of tumor growth and rescue of tumor cells from apoptosis (77,78). CXCR7 was recently identified as a receptor that affects tumor cell survival (79). SDF1α was shown to mediate the homing of hematologic stem cells to the bone marrow via CXCR4 signaling (80). High expression of CXCR4 correlates with insensitivity against treatment with sunitinib in mRCC and thus represents a possible mediator of therapy resistance in tumors (81). In addition, evidence indicates that SDF1α/CXCR4 signaling induces EGFR activation in human trophoblast cells (82). Interestingly, a selective targeted therapy drug, plerixafor, against CXCR4 has been approved by the FDA for mobilization of hematopoietic stem cells from the bone marrow for collection from peripheral blood for autologous stem-cell transplantation in patients with non-Hodgkin’s lymphoma (NHL) or multiple myeloma (MM) (83). Plerixafor shows an excellent safety profile even when administered in combination with cytotoxic chemotherapy in cancer patients (84). Most importantly, in an experimental setting, plerixafor was shown to be effective for the treatment of metastatic lung cancer, including NSCLC (72) and in inhibition of invasiveness of human CRC cells (85).

### EGFR-EGF

A further candidate of a targeted therapy multi-drug regimen is the EGFR oncogene pathway. Evidence for the increased expression of EGF in tumors treated with VEGFR inhibitors has been provided (30). As described above, inhibitors against EGFR signaling have already been approved for the treatment of EGFR-activated NSCLCs (19). Furthermore EGFR signal transduction steers similar intracellular signaling cascades such as the SDF1α/CXCR4 axis, PI3K/Akt and MAPK (ERK1/2) (17) and thus may also be involved in drug resistance. In addition, EGFR was found to increase the expression of angiogenic factors such as the enzyme cyclooxygenease 2 (COX2) (86).

### COX2-E-prostanoid receptors (EP)

The COX2 metabolic pathway results in the production of prostaglandin E2 (PGE2) which activates G-protein coupled EP. COX2 is increased in cancer and other pathological conditions and is suspected to participate in carcinogenesis and in tumor angiogenesis. It was shown that the COX2 pathway indirectly induces the upregulation of VEGF via the PKC pathway in NSCLC and in human lung fibroblasts (87,88). COX2 was reported to increase the expression of SDF1α and CXCR4 via PGE2 in myeloid-derived suppressor cells in ascites from ovarian cancer patients (89) and that it contributes to cell survival of human bladder cancer cells (90). In an experimental setting, the selective inhibition of COX2 reduced the growth of colon carcinoma cells *in vivo*(91). In malignant gliomas, COX2 inhibitors are currently been discussed for use in cancer treatment (92). Several selective targeted therapy drugs against COX2 [such as celecoxib (Celebrex) and etoricoxib (Arcoxia)] have been approved for the treatment of arthritis, osteoarthritis, dysmenorrhea and acute pain. The possible involvement in tumor development and the connection between COX2 signaling and the induction of VEGF, SDF1α and CXCR4 qualifies this enzyme as a potential candidate for a multi-drug regimen therapy for cancer treatment.

**Figure 1:**
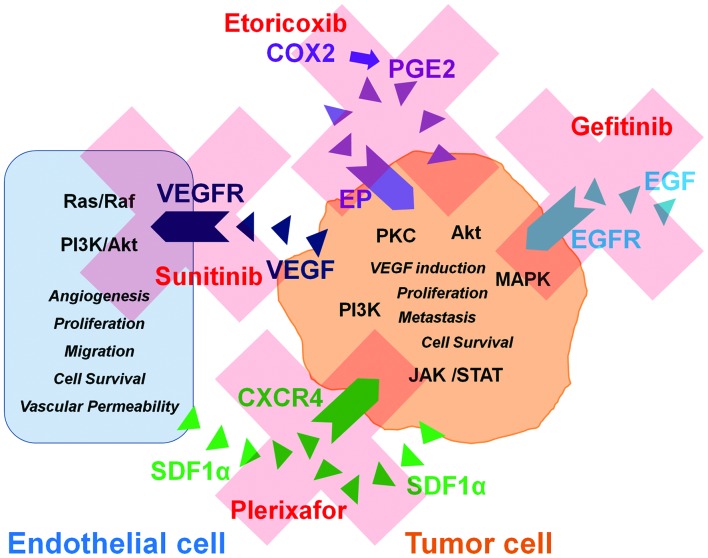
Hypothesis of overcoming the resistance to cancer treatment by the inhibition of different signaling pathways involved in non-small cell lung cancer (NSCLC) tumor growth by a multi-drug regimen of targeted therapy drugs. Intracellular signaling pathways activated by VEGFR, EGFR, CXCR4 and E-prostanoid receptors (EP) in tumor cells and in endothelial cells found to be involved in NSCLC tumor growth and maintenance are shown. Recent observations indicate the existence of crosstalk mechanisms between several of these pathways leading to resistance against single-agent targeted therapies alone or in combination with chemotherapeutics. Multiple inhibition of intracellular connected pathways may overcome the tumor insensitivity for targeted therapies. Targeted therapy drugs such as sunitinib, gefitinib, etoricoxib and plerixafor are clinically evaluated and FDA approved. PGE2, prostaglandin E2; VEGFR, vascular endothelial growth factor receptor; EGFR, epidermal growth factor receptor; COX2, cyclooxygenase 2.

The combined use of inhibitors against VEGFR signaling, such as sunitinib or bevacizumab combined with the CXCR4 inhibitor plerixafor and an inhibitor of EGFR signaling such as gefitinib and the COX2 inhibitor etoricoxib would block multiple signaling pathways in NSCLC. These pathways would be blocked at the receptor level while inhibiting multiple intracellular connected downstream pathways involved in tumor development and treatment resistance (Ras/Raf, PI3K/Akt, Jak/Stat, MAPK, PKC) (Fig. 1). Thus, the simultaneous inhibition of intracellular connected pathways in NSCLC tumors may overcome resistance mechanisms to targeted therapy drugs commonly noted in monotherapies or in a combination of targeted therapies with chemotherapeutics. All of the mentioned drugs are approved for different indications, and therefore their clinical profiles such as pharmacokinetics, pharmacodynamics and toxicities are well known. They are readily available as study drugs for usage in a clinical trial.

In order to identify NSCLC patient subgroups that may respond to this therapeutic concept, inclusion criteria should require the proof of expression of the respective targets. Bronchoscopy is a standard diagnostic procedure in NSCLC that allows biopsy of tumor tissue. The collected tumor specimens can easily be used for mRNA expression analysis of the respective targets by specific RT-real time PCR or by a customized gene expression array.

Similar to treatment of HIV by HAART, the herein proposed combination of targeted therapy drugs for antitumor treatment would fulfill the requirement of i) being highly selective and ii) inhibiting multiple targets involved in disease mechanism simultaneously. This may offer the chance for NSCLC patients beyond treatment to achieve an antitumor effect.
